# Assessment of Technetium-99m Labeled Macroaggregated Albumin Rhinoscintigraphy for the Measurement of Nasal Mucociliary Transport Rate: Intratest, Interobserver, and Intraobserver Reproducibility

**DOI:** 10.1155/2014/982515

**Published:** 2014-03-05

**Authors:** Zeki Dostbil, Yusuf Dag, Ozlem Cetinkaya, Mehmet Akdag, Bekir Tasdemir

**Affiliations:** ^1^Department of Nuclear Medicine, Faculty of Medicine, Dicle University, 21280 Diyarbakir, Turkey; ^2^Department of Nuclear Medicine, Faculty of Medicine, Harran University, 63300 Sanliurfa, Turkey; ^3^Department of Otorhinolaryngology-Head and Neck Surgery, Faculty of Medicine, Dicle University, 21280 Diyarbakir, Turkey

## Abstract

*Objectives.* The measurement of mucociliary transport velocity by rhinoscintigraphy with Tc-99m-macroaggregated albumin (^99m^Tc-MAA) is reliable measure of mucociliary clearance. The aim of this study is to assess the intratest, interobserver, and intraobserver reproducibility of nasal mucociliary transport rate (NMTR) measurement. *Materials and Methods.* Twenty-two subjects were evaluated to determine intratest reproducibility and a group of 35 subjects was examined to determine inter- and intraobserver reproducibility. Rhinoscintigraphy with ^99m^Tc-MAA was used to measure NMTR in all study subjects. Paired NMTR measurements were compared using a range of statistical methodologies. Intraclass correlation coefficients (ICC) and repeatability coefficients and Bland-Altman plots were applied to assess the degree of intratest, interobserver, and intraobserver variation. *Results.* Statistical analysis of test and retest experiments demonstrated the statistical equivalence of intratest NMTR measurements, interobserver NMTR measurements, and intraobserver NMTR measurements. The intratest ICC, interobserver ICC, and intraobserver ICC were 0.96, 0.83, and 0.91, respectively, indicating that intratest and intraobserver reproducibility are excellent and interobserver reproducibility is good. *Conclusions.* Rhinoscintigraphy using ^99m^Tc-MAA results in highly reproducible measurement of NMTR. The use of radionuclide imaging in measuring NMTR results in excellent intratest and intraobserver reproducibility and good interobserver reliability.

## 1. Introduction

Nasal mucociliary transport is a physiological process in which the mucus layer of ciliated respiratory epithelium generates movement of particles towards the nasopharynx. Mucociliary transport is an essential protective response against inhaled foreign particulate matter. Functional mucociliary transport results in the entrapment of inhaled foreign particles and their conveyance towards the nasopharynx. This phenomenon is dependent upon the number of functional cilia, beat frequency, and the coordinated movement of the cilia, as well as the secretion of viscoelastic nasal fluid and other factors [1]. Foreign particles and inhaled microorganisms are filtered at the airway mucosa by means of nasal mucociliary transport [1, 2].

A variety of techniques are available to evaluate ciliary activity in the nasal mucosa. Stroboscopy, roentgenography, and photoelectron techniques can be used to evaluate ciliary activity and ciliary beat frequency. However, many such techniques are not ideally suited for routine clinical use [3]. The most widely utilized procedures for the evaluation of nasal mucociliary activity are rhinoscintigraphy and the saccharin test, with the latter having the advantages of simplicity and low cost. The main disadvantage of the saccharin test is that the nasal mucociliary transport rate (NMTR) cannot be measured directly and the outcome of the test relies on the patient's subjective sense of taste. Rhinoscintigraphy can generate objective and precise data that facilitates the quantitative analyses of NMTR [4]. Among researches, Tc-99m-macroaggregated albumin (MAA) is widely used for rhinoscintigraphy [3]. Many studies have demonstrated that rhinoscintigraphy is an objective and sensitive method used in the follow-up of nasal and paranasal surgery and for determining the effectiveness of pharmacologic therapy in various nasal pathologies [3, 5–9].

The measurement of mucociliary transport velocity by rhinoscintigraphy with ^99m^Tc-MAA is considered a reliable measure of mucociliary clearance by many physicians [3, 10]. However, no previous study has directly assessed the reproducibility of rhinoscintigraphy with ^99m^Tc-MAA in human patients. Therefore, we aimed to evaluate intratest, interobserver, and intraobserver reproducibility by rhinoscintigraphy using ^99m^Tc-MAA for the measurement of NMTR.

## 2. Materials and Methods

### 2.1. Study Population

Twenty-two patients were evaluated to determine intratest reproducibility and a group of 35 patients was examined to determine inter- and intraobserver reproducibility. The study group included healthy subjects and subjects with conditions known to impact NMTR including tobacco use, allergic rhinitis, nasal septal deviation, nasal polyposis, turbinate hypertrophy, a history of any chronic disease, and a history of nasal airway surgery. The criteria for exclusion were as follows: nasal obstruction, current nasal drainage, cystic fibrosis, or history of cilia-related disorders. Prior to the study, an ethical review was conducted by the ethics committee of our institution and all study protocols were approved by the committee. Written informed consent was obtained from all subjects. All subjects were examined by an ear, nose, and throat specialist who provided detailed ear, nose, and throat evaluation.

### 2.2. Scintigraphic Procedure

Rhinoscintigraphy was performed by administering one droplet (~100 *μ*Ci that gives about 50 *μ*Sv radiation dose to the patients) of ^99m^Tc-macroaggregated albumin (^99m^Tc-MAA) (particle size: 10–150 *μ*m) on the right side, at the base of the nasal meatus and the anterior end of the inferior turbinate using a 27 G syringe. The scintigraphic procedure resulted in negligible gamma radiation exposure due to the small dosage of ^99m^Tc-MAA applied [11]. Room temperature was stabilized at 21°C. Images were obtained using a GE-millennium gamma camera system (GE Medical Systems, Milwaukee, WI, USA) with detectors set laterally with patients in the supine position. Thirty-second dynamic images were collected over a period of 20 minutes. To determine intratest reproducibility, the test was repeated after 4-hour interval on the same day using the same procedures and conditions. To determine interobserver and intraobserver reproducibility, NTMR imaging data were independently evaluated on at least two separate occasions. Following the test, the images were evaluated to calculate NMTR in millimetres per minute (mm/min). The distance between the point where the radiopharmaceutical was applied and the point at which the radioparticles reached the nasal cavity was measured as a straight line using the imaging system. This distance was divided by the time elapsed to calculate the NMTR in mm/min.

### 2.3. Statistical Analysis

Statistical analysis was performed using the SPSS 16.0 (SPSS Inc., Chicago, Illinois, USA) and MedCalc 12.7 (MedCalc, Mariakerke, Belgium) statistical software. Data are reported as mean ± standard deviation (SD). A paired *t*-test was used to compare NMTR measurements between observers 1 and 2 to determine interobserver reproducibility and to compare 1st and 2nd measurements by the same observer as a measurement of intraobserver reproducibility. The Wilcoxon signed rank test was applied in comparison of NMTR measurements on the 1st and 2nd scan, a measure of intratest reproducibility. A *P* value of less than 0.05 was set as the threshold of statistical significance.

Reliability refers to the degree to which a test is consistent and stable in measuring what it is intended to measure under identical conditions. A test is considered reliable if it is repeatable and reproducible within itself and across time and results in the ability to consistently obtain the same measurements. Intraobserver reliability is the ability of the same observer to obtain similar measurements consistently, whereas interobserver reliability is defined as the ability of different observer to obtain similar measurements under the same circumstances. Intratest reliability is the ability of a method to give similar measurements of the same subjects when measured by the same observer.

The intraclass correlation coefficient (ICC) estimates the overall correlation between all possible values within the measured variables. The intraclass correlation coefficient was used to quantify the degree of consensus between and among observers and between individual scans (a two-way mixed model, single measures, and consistency type was used). The possible values of ICC are between 0 and 1. Repeated measurements with an ICC value of 1 indicate perfect reproducibility, while a value of 0 represents reproducibility that is not better than that predicted by chance alone. An ICC value greater than0.75 is considered as reliable and clinically useful and repeated measurements with an ICC value lower than 0.7 are generally regarded as not clinically useful [12, 13].

The Bland-Altman plot (difference plot) is used to assess the repeatability of a method by comparing repeated measurements in a series of patients [14]. We calculated the coefficient of repeatability using Bland-Altman plots to evaluate the correlation between repeated measurements of NMTR. The mean difference should be zero if there is a perfect agreement between multiple measurements if the same method was used for repeated measurements. A positive value indicates that the same parameter measured on a second occasion resulted in a smaller value. In contrast, a negative value indicates that the same parameter measured on a second occasion resulted in a larger value. The repeatability coefficient is defined as 2 standard deviations of the mean differences between the repeated measurements. Therefore, 95% of repeated measurements for the sample will be within the range of mean difference ± the repeatability coefficient.

## 3. Results

The mean age was 26 ± 9.3 years in the intratest reproducibility trial and the mean age was 31.8 ± 10.2 years among the interobserver and intraobserver reproducibility subjects. The measured NMTRs for the intratest, interobserver, and intraobserver reproducibility subjects are shown in Table 1. No statistically significant difference in NMTR was found between the 1st and 2nd scan of the intratest group (*P* = 0.338), between observers 1 and 2 of the interobserver group (*P* = 0.211), or between the 1st and 2nd measurement of the intraobserver test group (*P* = 0.421). Nasal mucociliary transport rates measured for intratest, interobserver, and intraobserver reliability and statistical comparison are reported in Table 1.

Intratest ICC, interobserver ICC, and intraobserver ICC were 0.96, 0.83, and 0.91, respectively. These values demonstrate that NMTR measurements were reproducible among all groups. Intratest and intraobserver testing evaluation revealed excellent reproducibility with ICCs greater than 0.90. It appears that NMTR measurements are more reliable in intratest and intraobserver applications than was demonstrated in the interobserver trial. Intratest reliability was greater than the intraobserver reliability (Table 2).

The repeatability coefficients for the intratest, interobserver, and intraobserver trial of NMTR measurement by the rhinoscintigraphy were 1.8, 3.95, and 2.9, respectively. This means that for any two measurements chosen at random the ratio of the larger measured value to the smaller measured value would be at most 1.8, 3.95, or 2.9, respectively. Like ICC, the repeatability coefficient for the intratest and intraobserver measurements was lower than the repeatability coefficient of the interobserver measurements. This means that the intratest and intraobserver agreement was greater than the interobserver agreement among NMTR measurements. Similarly to the data discussed above, intratest agreement was greater than the intraobserver agreement.

## 4. Discussion

The present study demonstrates that the NMTR measured using repeated ^99m^Tc-MAA rhinoscintigraphy twice in a 4-hour interval on the same day did not differ significantly between measurements (*P* = 0.338). Similarly, NMTR did not differ significantly when calculated by two different nuclear medicine specialists on the same patient image (*P* = 0.211) and two NMTRs calculated by the same specialist at two different time points on the same images did not differ significantly (*P* = 0.421) (Table 1). Statistical comparison has demonstrated that two independent sets of measurements are statistically identical in all three reproducibility groups. NMTR measurements by rhinoscintigraphy are a reproducible method in intratest, interobserver, and intraobserver applications.

The intraclass correlation coefficient values for intratest, interobserver, and intraobserver reproducibility were 0.96, 0.83, and 0.91, respectively, in the reliability analysis. Here, variation in NMTR measurements varied less when recorded by the same observer than when measured by different observers. That is, rhinoscintigraphy has greater intraobserver reproducibility than interobserver reproducibility. This was an expected result. The interobserver ICC was greater than 0.75, regarded as good. However, it should be taken into account that the two observers participating in this study were working at the same center. Each was familiar with the processing patterns of the other observer. If NMTR is calculated by different observers working at different centers, it is possible to speculate that the measurements might be expected to vary by a greater degree than that observed in our study. This emphasizes the lack of standardization in processing methods for images measuring NMTR.

We initially hypothesized that the intraobserver reproducibility of the test would be greater than the intratest reproducibility. However, we found the opposite. Intratest ICC was slightly greater than the intraobserver ICC. During the NMTR measurements for the intratest reproducibility, the same observer processed the test and retest images consecutively. However, for the intraobserver reproducibility trials, we evaluated the images at different time points. The increased time interval between the processing of the two images may account for the lower ICC in the intraobserver group relative to our observation for the intratest group.

**Figure 1:**
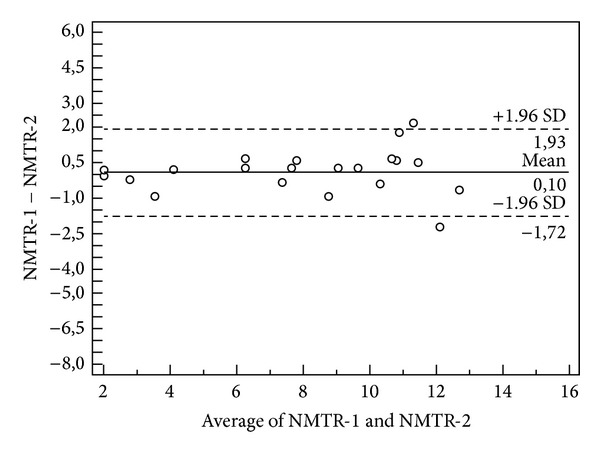
A Bland-Altman plot for intratest agreement is shown. The data presented here are NMTR measurements in two rhinoscintigraphy scans in the same subject. The mean difference and 95% confidence interval are indicated on the graph. The *y*-axis indicates the difference between the two measures (initial, NMTR and second scan, NMTR) and the *x*-axis indicates the mean of the two measures.

**Figure 2:**
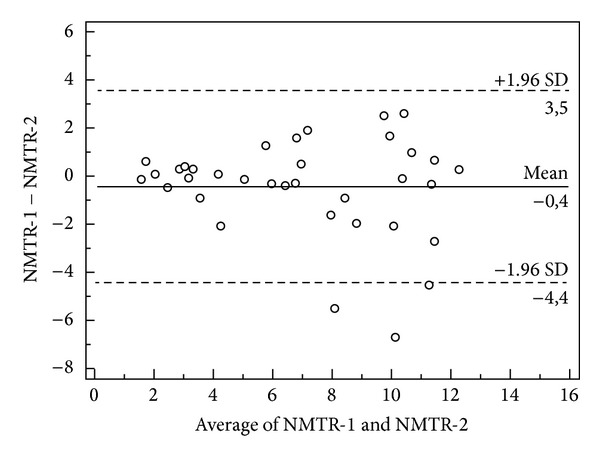
A Bland-Altman plot for interobserver agreement is shown. The data presented here are for NMTR measures by two different observers in the same scans. The mean difference and 95% confidence interval are indicated on the graph. The *y*-axis indicates the difference between the two measures (observer 1, NMTR and observer 2, NMTR) and the *x*-axis indicates the mean of the two measures.

**Figure 3:**
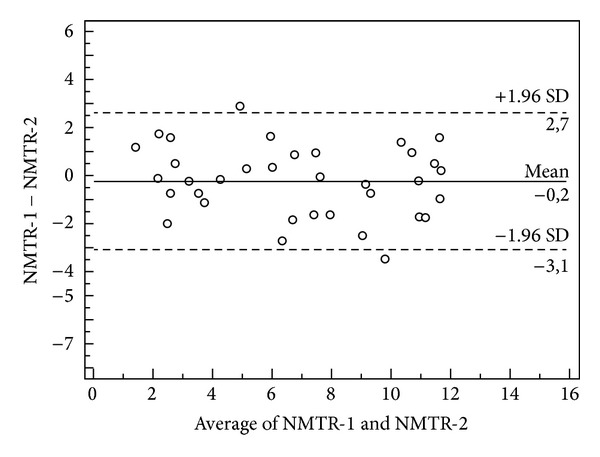
A Bland-Altman plot for intraobserver agreement is shown. The data presented here are for repeated NMTR measures by the same observer. The mean difference and 95% confidence interval are indicated on the graph. The *y*-axis indicates the difference between the two measures (first and second, NMTR) and the *x*-axis indicates the mean of the two measures.

As shown in the Bland-Altman plots, the three mean difference values in the test-retest, two independent observers, and repeated measurements trials were all approaching zero for all measured variables (Figures 1, 2, and 3). The repeatability coefficients and Bland-Altman plots were consistent with the ICC analysis.

In conclusion, the results of our study have demonstrated an excellent degree of intratest and intraobserver reproducibility and good interobserver reproducibility for the measurement of mucociliary transport velocity by rhinoscintigraphy with Tc-99m-macroaggregated albumin (^99m^Tc-MAA). Our results indicate that rhinoscintigraphy is a highly reliable method for the measurement of NMTR. Thus, this technique may be used reliably by researchers to measure NMTR in the nasal cavity.

## Figures and Tables

**Table 1:** Nasal mucociliary transport rates measured for intratest, interobserver, and intraobserver reproducibility and statistical comparison.

Type of reliability	NMTR (mm/min)		*P* value
	Mean ± SD	Range	
Intratest (*n* = 22)			
1st scan	8 ± 3.4	2–12.4	0.338
2nd scan	7.9 ± 3.3	1.9–13.2	
Interobserver (*n* = 35)			
Observer 1	6.8 ± 3.3	1.5–12.4	0.211
Observer 2	7.2 ± 3.7	1.4–13.5	
Intraobserver (*n* = 35)			
1st measurement	6.8 ± 3.3	1.5–12.4	0.421
2nd measurement	7 ± 3.6	0.8–12.1	

NMTR: nasal mucociliary transport rate.

**Table 2:** The intratest, interobserver, and intraobserver intraclass correlation coefficients (ICC) with 95% confidence intervals for the nasal mucociliary transport rates.

Type of reliability	ICC (95% CI)
Intratest (*n* = 22)	0.96 (0.916–0.985)
Interobserver (*n* = 35)	0.83 (0.693–0.911)
Intraobserver (*n* = 35)	0.91 (0.832–0.954)

ICC: intraclass correlation coefficient.
